# Surviving genocide in Srebrenica during the early childhood and adolescent personality

**DOI:** 10.3325/cmj.2013.54.55

**Published:** 2013-02

**Authors:** Nermina Kravić, Izet Pajević, Mevludin Hasanović

**Affiliations:** 1 Department of Psychiatry, Tuzla University Clinical Center, Tuzla, Bosnia and Herzegovina; 2 School of Medicine, Tuzla University, Tuzla, Bosnia and Herzegovina

## Abstract

**Aim:**

To examine how the experience of genocide in Srebrenica in the early childhood (ages 1-5) influences the psychological health in adolescence.

**Methods:**

This study included 100 school-attending adolescents, age 15-16 (born in 1990-91) who were divided in two groups according to the place of residence from 1992-1995: the Srebrenica group – adolescents who lived in Srebrenica during the siege and the non-Srebrenica group who lived in the “free territory,” were not wounded, and experienced no losses. We used the socio-demographic questionnaire created for the purposes of our study and the War Trauma Questionnaire, Posttraumatic Stress Reactions Questionnaire, Self-report Depressive Scale (Zung), Freiburg Personality Inventory, and the Lifestyle Questionnaire.

**Results:**

Srebrenica adolescents experienced significantly more traumatic experiences (14.26 ± 3.11 vs 4.86 ± 3.16, *P* < 0.001). Although there was no significant difference in the total score of posttraumatic stress reactions and intensity of depression between the two groups, significantly higher scores of posttraumatic stress reaction were noticed for several specific questions. The most prominent defense mechanisms in both groups were projection, intellectualization, and reactive formation. Srebrenica adolescents had higher sociability levels (34.7% vs 16.0%, χ^2^ = 7.231, *P* = 0.020).

**Conclusion:**

Srebrenica adolescents reported significantly more severe PTSD symptoms and significantly greater sociability. Our findings could be used for planning treatment and improving communication and overcoming traumas in war-affected areas.

Stressful early life experiences of war have been shown to influence behavioral, emotional, and cognitive development of children and shape their brain morphology (1,2).

During the 1992-95 war in Bosnia and Herzegovina, more than 100 000 people died or went missing (3). The town Srebrenica, United Nation’s “safe haven” area, was held siege by Serbian military forces for more than three years. After the fall of the town, over 8000 captured boys and men were premeditatedly and systematically killed, and 20 000 women, children and the elderly were forced to leave their homes. In 2008, the International Court of Justice declared the massacre genocide (4). The bodies of the victims are still being exhumed from the mass gravesites (5).

The impact of trauma and coping skills vary according to maturity and experience of the child, especially according to the degree of reliance on parents, adult caretakers, siblings, and peers (6).

Studies of children and adolescents from Bosnia and Herzegovina (7-13), Croatia (14,15), Cambodia (16), and Israel and Palestine (17) showed that growing up and living in war conditions increased anxiety, depression, and PTSD symptoms, but also most of children, in traumatic situations, enhanced the ways of active coping with intellectual and emotional focusing (18).

Our aims were to examine the influence of war traumatic experiences from the early childhood (age 1-5) on psychological health during adolescence, and the relationship between traumatic experiences, development of personality, and psychological defense mechanisms that influence adaptation and growth.

## Participants and methods

### Participants

The sample consisted of 100 school attending adolescents, boys and girls, aged 15-16 years, born in 1990-1991, who were 1-5 years old during the war. The participants were divided in two groups according to the place of residence during the war – the Srebrenica group consisting of 50 adolescents who were in Srebrenica and the non-Srebrenica control group consisting of 50 adolescents who were born and lived in the free territory of Tuzla area and were exposed to certain war traumas, but did not have any intensive traumatic experiences (wounding, loss of parent/s or sibling/s). All participants lived in the Tuzla area in the time of testing. The groups were selected according to their place of origin obtained from the school records and confirmed by direct contact with the adolescents, teachers, parents, and care takers. Ethical approval was obtained from the Ethics Committee of the University Clinical Centre Tuzla, Department for Education and Pedagogy, and verbal consent was obtained from parents/care takers, and adolescents. The research was conducted in the spring of 2006 in association with local schools, refugee settlements, and an SOS village for orphan children in the Tuzla area. The adolescents were tested in schools and classrooms together with other students and supervised by competent staff – a psychologist, psychiatrist, or teacher. The adolescents were told that they could stop answering the questions at any time if it became too disturbing for them.

### Data collection

The questionnaire on basic personal and socio-demographic information was designed specifically for this study and included questions on basic data (age, sex, family structure, and school success).

For war trauma and trauma reactions assessment we used the War Trauma Questionnaire and Posttraumatic Stress Reactions Questionnaire constructed by Stuvland and Djapic and UNICEF during the war in Bosnia and Herzegovina (18). The questionnaire contains descriptions of 28 war traumatic events and participants have to answer whether they were exposed to them with yes or no. The Posttraumatic Stress Reactions Questionnaire consists of 20 items and the participants answer how frequently they experienced a certain reaction: never – 1, sometimes – 2, or often – 3. This questionnaire assessed all three PTSD symptom clusters (hyper arousal, avoidance, and re-experiencing), as well as a fourth cluster of narrow range of feelings and alienation. The total result was the average of the answers to all 20 items.

Self-Rating Depression Scale (Zung) consists of 20 items scored for symptom intensity from 1 to 4. We evaluated the intensity of depression by adding a conversion table, which rated depression from euthymia (score below 50 points), minimal to mild depression (50-59 points), mild to developed (60-69 points), and heavy to extreme depression (70 points and more) (19).

Freiburg Personality Inventory (FPI), a multidimensional personality inventory, was used to quantify important personality traits. It consists of 212 statements which have to be answered with yes or no. It collects information about nine personality characteristics: neuroticism (psychosomatic complains), spontaneous aggressiveness, depression, anger, sociability, self-control, reactive aggressiveness (dominance), inhibition, openness (self criticism), and three added scales: extraversion/introversion, emotional stability/labiality, and masculinity. Results are presented in a range from 1 to 9 (1-3 low, 4-6 average, 7-9 high). The inventory was standardized for use in ex-Yugoslavia countries by Bele-Potočnik (20).

The Lifestyle Questionnaire (LSQ) measures psychological defense mechanisms by quantifying behavior styles typical for certain defense mechanisms. The LSQ consists of 92 statements, which should be answered with yes or no. Statements are divided into eight categories: reaction formation, negation, regression, inhibition, compensation, projection, intellectualization, and displacement. Results are in the range from 0 to 100, with scores >60 indicating high results. The questionnaire was standardized for use in ex Yugoslavia by Lamovec (21).

The War Trauma Questionnaire and Posttraumatic Stress Reactions Questionnaire is appropriate for children aged 7-18 years and was used with the permission of the Department for Psychology, Faculty of Philosophy, University of Sarajevo. Self-Rating Depression Scale (Zung) (20), Freiburg Personality Inventory (FPI) (21), and Lifestyle Questionnaire (LSQ) (22) are appropriate for children aged 15-16 years. We did not have specific authors’ permission for their use, but our psychologists who interpreted the test results are permitted to use them in psychometric studies.

### Statistical analysis

For variables with normal distribution, results were presented as mean±standard deviation (SD) and for variables with non-parametric distribution we used Man Whitney U test. To test the differences between the groups, we used χ^2^ test, *t* test for interval variables, odds ratio for nominal variables, Pearson r coefficient for assessment of correlations between some characteristics, and the α coefficient to test reliability. The level of statistical significance was set at *P* < 0.05. All statistical analyses were performed with SPSS for Windows, version 10.0 (SPSS Inc., Chicago, IL, USA).

## Results

A total of 50 Srebrenica and 50 non-Srebrenica adolescents were investigated. The mean age of participants ± standard deviation was 15.66 ± 0.48 years. There were no significant differences between the groups according to age (χ^2^ = 0.178, *P* = 0.673) and sex (46.0% male and 54.0% female participants in the total sample, χ^2^ = 0.161, *P* = 0.688).

All participants reported the range of 0-20 out of 28 war traumatic experiences. The average number of war traumatic experiences in the total sample was 9.56 ± 5.66; the Srebrenica group had significantly more war trauma experiences (14.26 ± 3.11) than the non-Srebrenica group (4.86 ± 3.16) (Z = -8.177, *P* < 0.001, Mann-Whitney U test). Although adolescents from both groups experienced war traumas during their childhood, significantly more participants from Srebrenica were exposed to traumatic situations (Table 1). Significantly more adolescents from Srebrenica experienced 17 of 28 traumatic experiences than adolescents from non-Srebrenica group (Table 1). There was no significant difference between the groups on the item “Close family member was in the army as a soldier” (92% from the Srebrenica group and 84% from the non-Srebrenica group) (Table 1).

**Table 1:** Frequencies of war traumatic experiences in Srebrenica (n = 50) and non-Srebrenica group (n = 50) of adolescents in postwar Bosnia and Herzegovina

	No. (%) participants in			
Traumatic experiences in the war	Srebrenica group	Non Srebrenica group	Odds ratio (95% confidence interval)*	*P*
	yes	yes		
Forced to leave my birth place because of the war	50 (100.0)	3 (6.0)	- (-)	<0.001
Parents separated because of the war for more than three weeks	33 (66.0)	10 (20.0)	7.76 (3.13-19.23)	<0.001
I was separated from my family for more than three weeks because of the war	21 (42.0)	7 (14.0)	4.48 (1.67-11.81)	0.003
Experienced shelling nearby	45 (90.0)	25 (50.0)	9.00 (3.02-26.43)	<0.001
Was in a shelter for more than three weeks	26 (52.0)	10 (20.0)	4.33 (1.78-10.53)	0.001
Experienced gun shooting nearby	46 (92.0)	27 (54.0)	9.79 (3.06-31.35)	<0.001
Father or/and mother killed in the war	32 (64.0)	0 (0.0)	- (-)	<0.001
Siblings killed in the war	2 (4.0)	0 (0.0)	- (-)	0.477
Close family member was in the army as a soldier	46 (96.0)	42 (84.0)	2.19 (0.61-7.81)	*0.227*
Lost a good friend during the war	7 (14.0)	4 (8.0)	1.87 (0.51-6.85)	0.343
Lost things or animals I loved during the war	31 (62.0)	13 (26.0)	4.64 (1.98-10.88)	0.001
Family member wounded in the war	41 (82.0)	17 (34.0)	8.84 (3.49-22.39)	<0.001
Family member captured/ went missing during the war	45 (90.0)	7 (14.0)	55.28 (16.30-187.52)	<0.001
My home was attacked or shelled during the war	49 (98.0)	4 (8.0)	563.50 (60.72-5229.71)	<0.001
There was a massacre in the place where I lived during the war	46 (92.0)	6 (12.0)	84.33 (22.28-319.17)	<0.001
Watched wounding, killing, torturing on TV or in newspapers during the war	35 (70.0)	26 (52.0)	2.15 (0.95-4.89)	0.067
Witnessed killing or wounding	12 (24.0)	7 (14.0)	1.98 (0.63-5.43)	0.207
Witnessed rape or sexual abuse during the war	3 (6.0)	1 (2.0)	3.13 (0.31-31.14)	0.331
Heard sounds of torture and beating	25 (50.0)	3 (6.0)	15.67 (4.30-57.03)	<0.001
Helped or was with a wounded or killed person	4 (8.0)	5 (10.0)	0.78 (0.20-3.10)	0.727
Was under a direct threat of killing during the war	2 (4.0)	1 (2.0)	2.04 (0.18-23.26)	0.565
Witnessed killing or wounding	8 (16.0)	1 (2.0)	9.33 (1.12-77.70)	0.039
Thought I would die of cold during the war	28 (56.0)	8 (16.0)	6.68 (2.61-17.10)	<0.001
Thought I would starve during the war	36 (72.0)	5 (10.0)	22.14 (7.61-70.30)	<0.001
Shot by sniper	0 (0.0)	0 (0.0)	-	1.0
Thought I would be killed or die during the war	32 (64.0)	9 (18.0)	8.10 (3.21-20.40)	<0.001
Wounded in war	1 (2.0)	0 (0.0)	- (-)	1.0
Captured or in concentration camp during the war	3 (6.0)	0 (0.0)	- (-)	0.243

*Mantel-Haenszel common odds ratio.

There was no significant difference between two groups in posttraumatic stress reactions scores. Srebrenica group had a significantly higher posttraumatic stress reactions score for the symptoms “When I remember a traumatic event I feel very uncomfortable” (*t* = 2.45, *P* = 0.016), “Trying not to be surprised by some danger” (*t* = 2.35, *P* = 0.021), and “Difficulties with sleep” (*t* = 2.48, *P* = 0.015) (Table 2).

**Table 2:** Posttraumatic Stress Reactions score (10) in Srebrenica (n = 50) and non-Srebrenica group (n = 50) of adolescents in postwar Bosnia and Herzegovina

Posttraumatic stress reactions*	Srebrenica group		Non Srebrenica group		t	P*	
	mean	standard deviation	mean	standard deviation			
When remembering a traumatic event I feel very uncomfortable	2.34	(0.72)	2.00	(0.67)	2.45	0.016	
Trying not to think about it	2.58	(1.36)	2.24	(0.72)	1.57	0.212	
Trying to avoid everything what reminds me of it	2.42	(0.70)	2.38	(0.69)	0.29	0.776	
Trying not to be surprised by some form of danger	2.56	(0.58)	2.24	(0.77)	2.35	0.021	
I avoid talking about it	2.36	(0.75)	2.16	(0.76)	1.32	0.190
Since then, I don’t enjoy fun, sports and other things I used to do	1.60	(0.73)	1.44	(0.67)	1.14	0.257
Since then, I think nobody understands me	1.72	(0.81)	1.58	(0.70)	0.92	0.358
My feelings have changed (can't love, cry, I do not care about anything...)	1.54	(0.76)	1.50	(0.70)	0.27	0.786
My wishes about the future seem ineffective (what would I be, how long will I live, if am I going to have family...)	1.50	(0.65)	1.58	(0.73)	-0.58	0.564
I have difficulties with concentration	1.64	(0.66)	1.56	(0.67)	0.60	0.551
Pictures of that event appear in my mind	1.90	(0.76)	1.84	(0.81)	0.38	0.705
I have nightmares about that event	1.74	(0.80)	1.56	(0.70)	1.19	0.236
I think that it is happening again	1.44	(0.67)	1.46	(0.64)	-0.15	0.880
I feel like it was a dream	1.70	(0.61)	1.82	(0.72)	-0.89	0.372
It seems like it has never happened before	1.66	(0.71)	1.64	(0.66)	0.14	0.885
I can’t remember important parts of that event	1.82	(0.77)	1.84	(0.79)	-0,13	0.899
Difficulties with sleep	1.70	(0.81)	1.34	(0.62)	2.48	0.015
I get angry easily	1.88	(0.80)	1.94	(0.77)	-0.38	0.702
I have startle reactions	1.82	(0.75)	1.78	(0.68)	0.28	0.780
Remembering the event causes unpleasant body reactions (heart beating, sweating, ...)	1.98	(0.77)	1.80	(0.81)	1.14	0.257

*t test.

Non-Srebrenica group showed significantly better school success: 45 (90.0%) adolescents from the Srebrenica group and 35 (70%) from the non-Srebrenica group had excellent and very good marks, while 5 (10%) from the Srebrenica group and 0 (0%) from the non-Srebrenica group had bad marks (χ^2^ = 7.983, *P* = 0.046).

The α of reliability for Freiberg personality questionnaire (PFI) in the total sample was 0.91. For more than half of the adolescents the results for most personality characteristics were within the reference interval. For the characteristic openness about two thirds of adolescents from the Srebrenica group and more than half from the non-Srebrenica group showed a tendency toward having low values. The Srebrenica group showed a significantly higher level of sociability (χ^2^ = 7.231, *P* = 0.027) (Table 3). More than one third of the adolescents in both groups reported high levels of psychosomatic complains, spontaneous aggression, and reactive aggression (Table 3). There was no significant correlation between the number of traumatic experiences from the early childhood and personality profile characteristics.

**Table 3:** Personality characteristics in Srebrenica (n = 49*) and non Srebrenica group (n = 50) of adolescents in postwar Bosnia and Herzegovina measured by Freiburg Personality Questionnaire (12)

Personality characteristics	Group^†^	No. (%) of participants with the score			χ^2^	*P*^‡^
		low, 1-3	average, 4-6	high, 7-9		
Psychosomatic complains	S	5 (10.2)	25 (51.0)	19(38.8)	0.508	0.776
	NS	4 (8.0)	29 (58.0)	17 (34.0)		
Spontaneous aggression	S	2 (4.1)	31 (63.3)	16 (32.7)	0.834	0.659
	NS	1 (2.0)	29 (58.0)	20 (40.0)		
Depressiveness	S	3 (6.1)	32 (65.3)	14 (28.6)	0.024	0.988
	NS	3 (6.0)	32 (64.0)	15 (30.0)		
Irritability	S	8 (16.3)	36 (73.5)	5 (10.2)	2.724	0.256
	NS	3 (6.0)	42 (84.0)	5 (10.0)		
Sociability	S	2 (4.1)	30 (60.1)	17 (34.7)	7.231	0.027
	NS	0 (0.0)	42 (84.0)	8 (16.0)		
Control	S	1 (2.0)	41 (83.7)	7 (14.3)	2.644	0.267
	NS	4 (8.0)	36 (72.0)	10 (20.0)		
Reactive aggression	S	5 (10.2)	27 (55.1)	17 (34.7)	1.697	0.429
	NS	2 (4.0)	27 (54.0)	21 (42.0)		
Inhibition	S	8 (16.3)	31 (63.3)	10 (20.4)	3.543	0.170
	NS	7 (14.0)	39 (78.0)	4 (14.1)		
Openness	S	31 (63.3)	17 (34.7)	1 (2.0)	1.784	0.410
	NS	28 (56.0)	22 (44.0)	0 (0.0)		
Extraversion	S	3 (6.1)	34 (69.4)	12 (24.5)	1.158	0.560
	NS	1 (2.0)	35 (70.0)	14 (28.0)		
Emotional insecurity	S	5 (10.2)	36 (73.5)	8 (16.3)	0.161	0.923
	NS	6 (12.0)	37 (74.0)	7 (14.0)		
Masculinity	S	8 (16.3)	31 (63.3)	10 (20.4)	0.959	0.619
	NS	7 (14.0)	36 (72.0)	7 (14.0)		

*There was one invalid Freiburg Personality Questionnaire in Srebrenica group.

†S – Srebrenica group; NS – non Srebrenica group.

‡χ^2^ test.

The α coefficient for Lifestyle Questionnaire was 0.83. There were no significant differences between the two groups in defense mechanisms. Most adolescents from the Srebrenica group used projection (58.0%), intellectualization (54.0%), reactive formation (48.0%), and denial (40.0%), while adolescents from the non-Srebrenica group also used projection (72.0%), intellectualization (52.0%), and reactive formation (48.0%) as defensive attitude model (Table 4). There was no significant correlation between the number of traumatic experiences experienced in early childhood and the defense mechanisms used.

**Table 4:** Defense mechanisms in Srebrenica (n = 50) and non-Srebrenica group (n = 50) of adolescents in postwar Bosnia and Herzegovina measured with Lifestyle Questionnaire (13)

		No. (%) of participants who answered			
Defense mechanisms	Group*	no	yes	Odds ratio (95% confidence interval)†	*P*
Denial	S	30 (60.0)	20 (40.0)	1.41 (0.62-3.22)	0.405
	NS	34 (68.0)	16 (32.0)		
Suppression	S	45 (90.0)	5 (10.0)	2.66 (0.49-14.44)	0.255
	NS	48 (96.0)	2 (4.0)		
Regression	S	42 (84.0)	8 (16.0)	1.71 (0.519-5.66)	0.177
	NS	45 (90.0)	5 (10.0)		
Compensation	S	40 (80.0)	10 (20.0)	0.71 (0.28-1.82)	0.477
	NS	37 (74.0)	13 (26.0)		
Projection	S	21 (42.0)	29 (58.0)	0.53 (0.23-1.23)	0.144
	NS	14 (28.0)	36 (72.0)		
Displacement	S	44 (88.0)	6 (12.0)	1.22 (0.35-4.31)	0.750
	NS	45 (90.0)	5 (10.0)		
Intellectualization	S	23 (46.0)	27 (54.0)	1.08 (0.49-2.38)	0.841
	NS	24 (48.0)	26 (52.0)		
Reactive formation	S	26 (52.0)	24 (48.0)	1.00 (0.45-2.19)	1.000
	NS	26 (52.0)	24 (48.0)		

*S – Srebrenica group; NS – non Srebrenica group.

†Mantel-Haenszel common odds ratio.

The majority of adolescents from the Srebrenica group (51.0%) and the non-Srebrenica group (49.0%) had normal results on the Zung Self-Rating Depression Scale. The mean depression score in the Srebrenica group was at the low end of the minimal to middle range 50.13 ± 12.43 and in the non-Srebrenica group it was at the higher end of normal results 49.51 ± 10.85, and there was no significant difference between the groups (*t* = 0.26, *P* = 0.79) (Table 5). There was no significant correlation between the number of traumatic experiences experienced during the war and the level of depression (Pearson *r* - 0.048, *P* = 0.638).

**Table 5:** Depressiveness symptoms in Srebrenica (n = 49*) and non-Srebrenica group (n = 48*) of adolescents in postwar Bosnia and Herzegovina measured with Self Rating Depression Scale (Zung) (11)

		No. (%) of participants who answered			
Symptoms of depressiveness	Group^†^	no (n%)	yes (n%)	Odds ratio (95% confidence interval)^‡^	*P*
I feel downhearted and blue	S	44 (89.8)	5 (10.2)	0.79 (0.22-2.8)	0.722
	NS	42 (87.5)	6 (12.5)		
I feel the best in the morning	S	25 (51.0)	24 (49.0)	0.62 (0.28-1.41)	0.259
	NS	19 (39.6)	29 (60.4)		
I have crying spells or feel like it	S	43 (87.7)	6 (12.2)	0.60 (0.19-1.85)	0.379
	NS	39 (81.25)	9 (18.75)		
I have trouble sleeping at night	S	45 (91.8)	4 (8.2)	1.33 (0.28-6.3)	0.159
	NS	45 (93.75)	3 (6.25)		
I eat as much as I used to	S	18 (36.7)	31 (63.3)	0.78 (0.34-1.82)	0.569
	NS	15 (31.25)	33 (68.75)		
I like to look at and be in the company with attractive people	S	10 (20.4)	39 (79.6)	2.78 (1.13-6.86)	0.026
	NS	20 (41.7)	28 (58.3)		
I notice that I am loosing weight	S	45 (91.8)	4 (8.2)	0.62(0.16-2.36)	0.485
	NS	42 (87.5)	6 (12.5)		
I have trouble with constipation	S	44 (89.8)	5 (10.2)	0.000 (-)	0.071
	NS	48 (100.0)	0 (0.0)		
My heart beats faster than usual	S	44 (89.8)	5 (10.2)	0.79 (0.22-2.8)	0.722
	NS	42 (87.5)	6 (12.5)		
I get tired for no reason	S	40 (81.6)	9 (18.4)	2.47(0.71-8.66)	0.156
	NS	44 (91.7)	4 (8.3)		
My mind is as clear as it used to be	S	34 (69.4)	15 (30.6)	0.73 (0.32-1.71)	0.457
	*NS*	*30* (6*2.5)*	*18 (37.5)*		
I find it easy to do the things I used to	S	29 (59.2)	20 (40.8)	0.69 (0.31-1.54)	0.364
	NS	24 (50.0)	24 (50.0)		
I feel restless and I can’t keep still	S	40 (81.6)	9 (18.4)	1.31 (0.45-3.88)	0.616
	NS	41 (85.4)	7 (14.6)		
I feel hopeful about the future	S	37 (75.5)	12 (24.5)	0.49 (0.21-1.18)	0.114
	NS	29 (60.4)	19 (39.6)		
I am more irritable than usual	S	39 (79.6)	10 (20.4)	2.82 (0.82-9.72)	0.100
	NS	44 (91.7)	4 (8.3)		
I find it easy to make decisions	S	23 (46.9)	26 (53.1)	0.62 (0.27-1.40)	0.250
	NS	17 (35.4)	31 (64.6)		
I feel that I am useful and needed	S	33 (67.3)	16 (32.7)	0.48 (0.21-1.1)	0.085
	NS	24 (50.0)	24 (50.0)		
My life is pretty full	S	27 (55.1)	21 (44.9)	1.42 (0.62-3.22)	0.342
	NS	31 (64.6)	17 (35.4)		
It feel that others would be better of if I were dead	S	41 (83.7)	8 (16.3)	0.97 (0.33-2.85)	0.964
	NS	40 (83.3)	8 (16.7)		
I still enjoy the things I used to do	S	24 (49.0)	25 (51.0)	1.23 (0.55-2.73)	0.609
	NS	26 (54.2)	22 (45.8)		

*One Zung Scale in Srebrenica group and two in non-Srebrenica group were not completely filled out.

†S – Srebrenica group; NS – non Srebrenica group.

‡ Mantel-Haenszel common odds ratio.

There were significant negative correlations between the number of traumatic events experienced during the war and items on the Zung Self Rating Depression Scale “I feel the best in the morning” (Pearson r = -0.265, *P* = 0.009) and “I look at the future with confidence” (Pearson *r* = -0.234, *P* = 0.017). A positive significant correlation was found between the number of traumatic events and the item “I like to be in company of attractive people” (Pearson *r* = 0.221, *P* = 0.030).

## Discussion

The study found that the Srebrenica group experienced more intensive traumas but it did not find significant psychopathological differences between the Srebrenica and non-Srebrenica group.

More than 80% of adolescents from both groups reported that they had a close family member on the battlefield during the war. This fact could explain the lack of psychopathological differences between the groups since it has been shown that this factor has an indirect impact on psychical development (12). The lack of differences between groups can also be partly explained by the traumas of the other family members, which can be transmitted trans-generationally (22-24). All these factors indicate that our control group was not really protected from war traumas, which should be taken into account in the interpretation of the results.

The results of psychological characteristics of personality profiles on the FPI in our study were mostly within the reference range, except for openness. This was the only personality characteristic that had a low score in more than a half of the adolescents from both groups. This fact can be interpreted as a lack of confidence in others, which can be a result of war experiences (25). On the other hand, adolescents from the Srebrenica group had high values of sociability. It could be a protective and curative factor in the population of refugee adolescents, who usually live with family members and in refugee settlements with those who have experienced similar traumatic experiences (26).

High levels of psychosomatic complaints and spontaneous and reactive aggression were found in more than one third of adolescents from both groups, and in combination with low levels of openness could be interpreted as an incapability to put negative emotions into words and share them with others (27). These traits could increase the risk of psychosomatic illnesses and uncontrollable “acting out” behavior (28,29). Studies on adolescents who survived war trauma have shown different results. Khmer refugee adolescents who witnessed violence and met the criteria for PTSD, were mostly functioning adequately (30), while newly arrived refugee adolescents from Bosnia and Herzegovina in the US, who had experienced traumatic war violence, reported behavior problems (31).

The use of defense mechanisms is important in the process of forming the young person’s personality (32,33). We found no significant differences between the groups in psychological defense mechanisms, but both groups had high levels of projection, intellectualization, and reactive formation. This is understandable since young people have a need to convert negative experiences from early childhood and combine it with the natural process of maturing (34)

A US study showed repression, denial, and reactive formation to be dominant defensive mechanisms in adolescence but did not show an expected increase in intellectualization and a decrease in projection (35). The adolescents from our research showed increases in intellectualization as a more mature defense mechanism, but a high level of projection was also present, which could be interpreted as an attempt at adaptation and self-protection in difficult life circumstances.

The level of depression in both groups was mostly normal to minimal. We found that a high number of traumatic experiences correlated with negative answers for “feeling good in the morning” and “having confidence in the future,” which implies a lower long-term life motivation. Other studies found higher depression among those youths who blamed others for their parent’s suicide (36) and those with high exposure to rocket attacks and low social support (37). Negative psychosocial outcomes in children were also associated to parental involvement in the Croatian war 1991-1995, especially among boys (14). In our study, more war traumatized group had a significantly positive correlation with the desire to be in the company of attractive people, which is in agreement with a higher result for socialization.

In the war in Bosnia and Herzegovina, refugees, women, and children had access to psychological support (38). Therefore, these psycho-social interventions may have had long-time positive effect (39), which may also be a reason why we did not find a big difference between the two groups.

The study limitations included a relatively small sample, which yielded limited statistical power. Also, our research is based on self-report tests, and a more complete insight into the psychological health of adolescents may be obtained by collecting the assessments given by their parents/caregivers and teachers. However, it has been shown that adolescents’ self-rating scales gave more accurate results than interviews (40,41). Since the inhabitants of the entire Bosnia and Herzegovina were influenced by war, it was not possible to have a control sample entirely unaffected by war, which could also influence the results our research.

The children who survived traumatic war experiences might be more vulnerable for posttraumatic stress disorder and depression later in life (42). Therefore, it would be interesting to perform follow up studies on the same population, to assess the impact of war on the family, social, and professional functioning of the traumatized children.

The finding that a greater number of traumatic war experiences increases the level of sociability, could be used in treatment planning for adolescents, for example through group psychotherapy. Our findings could be used in the healing process of the restoration and reconciliation of a war traumatized society (43) and as a base for future psychosocial interventions in war-affected areas.
